# Carbonic Anhydrase IX Induces Human Osteosarcoma Cell Metastasis by Activating HSPA6 Expression Through the AMPK Signalling Pathway

**DOI:** 10.1111/jcmm.70763

**Published:** 2025-08-05

**Authors:** Jia‐Sin Yang, Chia‐Hsuan Chou, Yi‐Hsien Hsieh, Chih‐Hsin Tang, Ko‐Hsiu Lu, Shun‐Fa Yang

**Affiliations:** ^1^ Department of Medical Research Chung Shan Medical University Hospital Taichung Taiwan; ^2^ Institute of Medicine Chung Shan Medical University Taichung Taiwan; ^3^ Department of Pharmacology, School of Medicine China Medical University Taichung Taiwan; ^4^ Department of Medical Laboratory Science and Biotechnology Asia University Taichung Taiwan; ^5^ Chinese Medicine Research Center China Medical University Taichung Taiwan; ^6^ Department of Orthopedics Chung Shan Medical University Hospital Taichung Taiwan; ^7^ School of Medicine Chung Shan Medical University Taichung Taiwan

**Keywords:** AMPK, CAIX, HSPA6, metastasis, osteosarcoma

## Abstract

Osteosarcoma(OS), the most common primary bone cancer, has high metastatic potential and a high mortality rate. Carbonic anhydrase IX (CAIX), a hypoxia‐induced transmembrane protein, is highly expressed in numerous cancers. Over the past decades, scientists have made extensive efforts to determine the role of CAIX in various cancers. However, the effects of CAIX on the metastasis of OS cell lines remain unclear. We examined the effectiveness of CAIX for inducing the invasion and migration of human OS cells and the underlying molecular mechanisms. We established CAIX‐overexpressing human OS cells and found markedly increased migratory and invasive abilities in HOS and U2OS cell lines. In addition, CAIX overexpression increased the messenger ribonucleic acid (mRNA) and protein expression levels of heat shock protein family A member 6 (HSPA6) and the phosphorylation of adenosine‐monophosphate‐activated protein kinase (AMPK) signalling proteins. With HSPA6 knockdown, U2OS cells exhibited significantly reduced migratory and invasive abilities. Moreover, treatment with an AMPK inhibitor (dorsomorphin) suppressed CAIX‐induced HSPA6 expression and the metastasis of U2OS cells. In conclusion, the results indicate that CAIX overexpression mediates HSPA6 expression through the AMPK signalling pathway, which consequently induces the metastasis of OS cells.

## 1 Introduction

Osteosarcoma (OS) is the most common primary bone cancer, and its incidence is highest in adolescents aged 10–15 years and in older adults [1, 2, 3]. Approximately, six out of each one million children and two out of each one million adults are affected by OS [4]. In Taiwan, approximately 60 cases of OS are diagnosed each year [5]. OS commonly occurs in the metaphysis of long bones, including the distal femur, proximal tibia, and proximal humerus. The metaphysis contains growth plates that facilitate bone growth; thus, the incidence of OS may be related to the rate of bone growth [2]. The primary treatment for OS is surgery; however, the rate of survival for patients who undergo surgery alone is only 15%–17% [6]. Combining surgery with adjuvant chemotherapy can increase the 5‐year survival rate to nearly 70%. Despite major advances and improvements in medical treatment for OS, metastasis, chemotherapy resistance, and cancer recurrence often lead to treatment failure. Overall, 30%–40% of patients with OS exhibit local recurrence or distant metastasis [7], and the 5‐year survival rate in these patients is only 23%–29% [8]. Therefore, determining the mechanisms of cancer cell formation and metastasis as well as developing treatment strategies for reducing the mortality rate of OS are crucial [9, 10].

Carbonic anhydrases (CAs) are zinc metalloenzymes that catalyse the reversible hydration of carbon dioxide and water into bicarbonate and protons, which maintain the pH balance [11]. CAs may play a role in various physiological processes, including the transport of carbon dioxide and solutes as well as the acidification of the microenvironment; they may thus modulate the malignant phenotype of cancer cells [12, 13]. Carbonic anhydrase IX (CAIX) belongs to the alpha family of CAs and consists of signal peptides (SPs, removed during protein maturation), a proteoglycan (PG)‐like region, a CA (with a highly conserved active site), transmembrane regions, and intracellular tails [14, 15]. CAIX expression is induced under hypoxic conditions in various solid tumours, including renal cell carcinoma and breast, non‐small‐cell lung, ovarian, oesophageal, gastric, and cervical cancers [16, 17, 18, 19, 20, 21, 22, 23]. Hypoxia‐inducible factor‐1 binds to a hypoxia‐response element in the basal promoter of its gene (*CA9*), mediating the transcription of the *CA9* gene and subsequently leading to increased CAIX synthesis [24]. Numerous studies have highlighted the role of CAIX expression in promoting metastatic behaviour through diverse mechanisms, including extracellular acidification, modulation of cell adhesion and migration, and activation of pro‐metastatic signalling pathways [25, 26, 27, 28, 29]. However, no study has examined whether CAIX directly regulates OS and the underlying molecular mechanisms. The present study investigated how CAIX regulates the metastasis of OS cells and examined the detailed molecular mechanisms involved.

## 2 Materials and Methods

### 2.1 Cell Lines and Cell Culture

Several OS cell lines (143B, G292, HOS, MG‐63, Saos2 and U2OS) were cultured in different media. 143B and U2OS cells were cultured in Dulbecco's modified Eagle medium. G292 cells were cultured in McCoy's 5A medium. HOS and MG‐63 cells were cultured in minimum essential medium. Saos2 cells were cultured in Roswell Park Memorial Institute 1640 medium. All media were supplemented with appropriate antibiotics and 10% fetal bovine serum. After the overexpression or knockdown of specific genes, the cells were maintained at 37°C in a humidified atmosphere with 5% CO_2_ for a specified period before being harvested for subsequent experiments.

### 2.2 Transient Transfection

The HOS and U2OS cells were transfected with the pcDNA3.0‐CAIX or pcDNA3.0 vector by using Lipofectamine 2000 (Invitrogen, Life Technologies, Carlsbad, CA, USA) in accordance with the manufacturer's instructions. siRNA transfection was performed using Lipofectamine RNAiMAX (Invitrogen, Life Technologies, Carlsbad, CA, USA) in accordance with the manufacturer's instructions. After transfection for 24–48 h, the cells were used for the following experiments. The collected cells were processed for western blot analysis and a cell migration assay.

### 2.3 Reverse Transcription–Polymerase Chain Reaction and Real‐Time Quantitative Polymerase Chain Reaction

Total RNAs were isolated from OS cells by using the total RNA mini kit (Geneaid Biotech Ltd., Sijhih City, Taiwan). The isolated total RNAs (2 μg) were reverse‐transcribed into cDNAs by using the high‐capacity cDNA reverse transcription kit (Applied Biosystems, Foster City, CA, USA). Quantitative real‐time PCR was conducted using the Taqman qPCR master mix (Applied Biosystems, Foster City, CA, USA) or SYBR Green qPCR master mix (Applied Biosystems, Foster City, CA, USA) on a StepOnePlus sequence detection system [30]. For this PCR, 100 ng of total cDNA was added per 20‐μL reaction mixture containing SYBR Green reagents or Taqman probes. The experiments were performed in triplicate, and the relative levels of gene expression were calculated as Δ*C*_*t*_ = (*C*_*t*Gene_ − *C*_*t*Reference_). The fold change of gene expression levels was determined using the 2^(−ΔΔ*Ct*)^ method.

### 2.4 Western Blot Analysis

Cell lysates were separated through 10% sodium dodecyl sulfate–polyacrylamide gel electrophoresis. The lysates were transferred onto nitrocellulose membranes. The blots were subsequently incubated for 1 h with 5% bovine serum albumin in Tris‐buffered saline (20 mM Tris and 137 mM NaCl, pH 7.6) containing 0.1% Tween‐20 (TBS‐T) to block nonspecific binding, and the blots were probed with primary antibodies. The blots were then incubated with appropriate horseradish‐peroxidase‐conjugated secondary antibodies for 2 h. Signals were measured using an enhanced chemiluminescence commercial kit (Millipore, Bedford, MA, USA).

### 2.5 Boyden Chamber Assay

Cells were harvested and seeded at 10^4^ cells/well in a Boyden chamber (Neuro Probe, Cabin John, MD, USA) containing serum‐free medium, after which they were incubated for 24 h at 37°C. For the invasion assay, 10 μL of Matrigel (25 mg/50 mL; BD Biosciences, MA, USA) was applied to 8‐μm polycarbonate membrane filters [31]. The filters were then air‐dried for 3–5 h in a laminar flow hood. The invaded cells were fixed with methanol and stained with Giemsa stain. Cell numbers were counted under a light microscope. The migration assay was conducted following the same procedure as that applied for the invasion assay but without the coating of Matrigel.

### 2.6 Cell Cycle Analysis (Flow Cytometry)

Cells were harvested with trypsin–ethylenediaminetetraacetic acid, washed with phosphate‐buffered saline twice, and fixed in 70% ethanol at −20°C overnight. The fixed cells were pelleted and stained with propidium iodide in the dark for 15 min. Their DNA content was analysed using the flow cytometer Accuri C6 (BD Bioscience) [32].

### 2.7 Statistical Analysis

Values are expressed as mean ± standard deviation. The statistical significance of differences was determined using Student's *t* test (Sigma‐Stat 2.0, Jandel Scientific, San Rafael, CA, USA). A difference with *p* < 0.05 was considered statistically significant.

## 3 Results

### 3.1 CAIX Expression in Human OS 143B, G292, HOS, MG‐63, Saos2 and U2OS Cells

**FIGURE 1:**
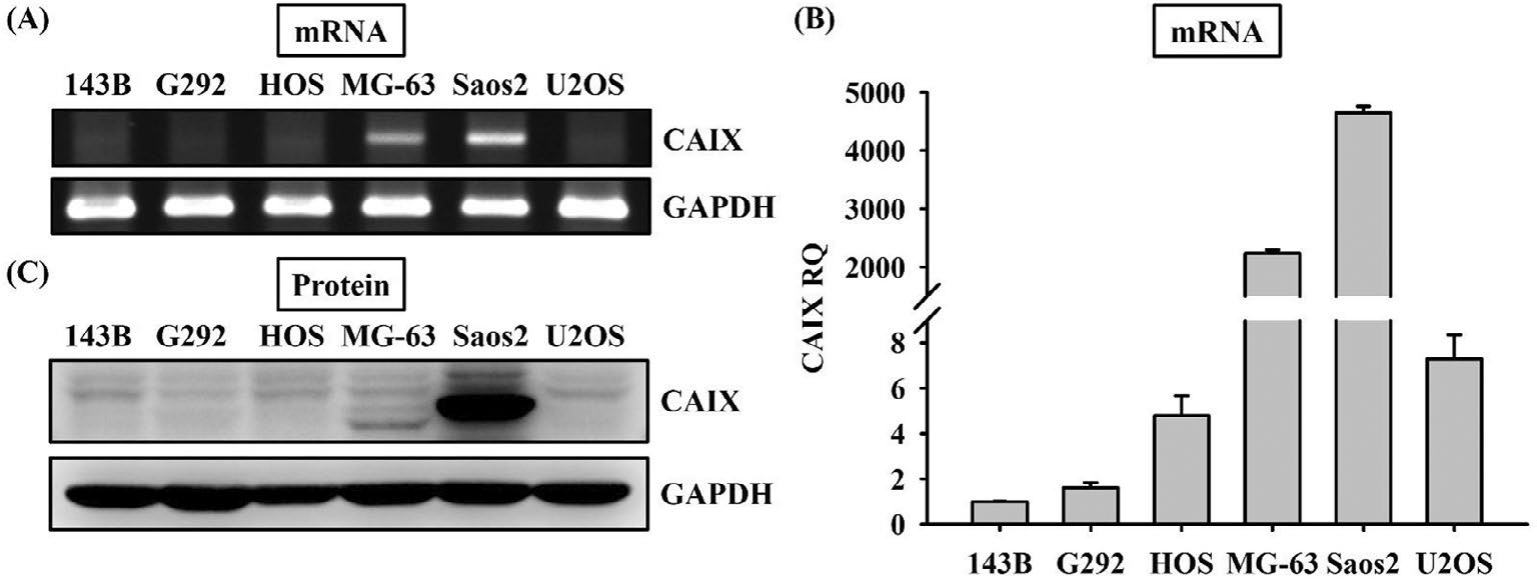
Expression of CAIX in human osteosarcoma cell lines. (A) Using RT‐PCR, (B) real‐time PCR and (C) western blot analysis, the CAIX mRNA and protein expressions in human osteosarcoma 143B, G292, HOS, MG‐63, Saos2 and U2OS cells were detected, respectively.

To assess CAIX expression in human OS 143B, G292, HOS, MG‐63, Saos2 and U2OS cells, western blot analysis, RT‐PCR and real‐time PCR were conducted. As expected, our results demonstrated inconsistent CAIX mRNA and protein expression levels in the different cell lines. Lower CAIX mRNA and protein expression levels were observed in 143B, G292, HOS, and U2OS cells, and the highest expression levels were discovered in MG‐63 and Saos2 cells (Figure 1A–C). Accordingly, we selected HOS and U2OS cells for all subsequent experiments to investigate the modulation of OS metastasis by CAIX and the underlying mechanisms.

### 3.2 CAIX Induces HOS and U2OS Cell Migration and Invasion

**FIGURE 2:**
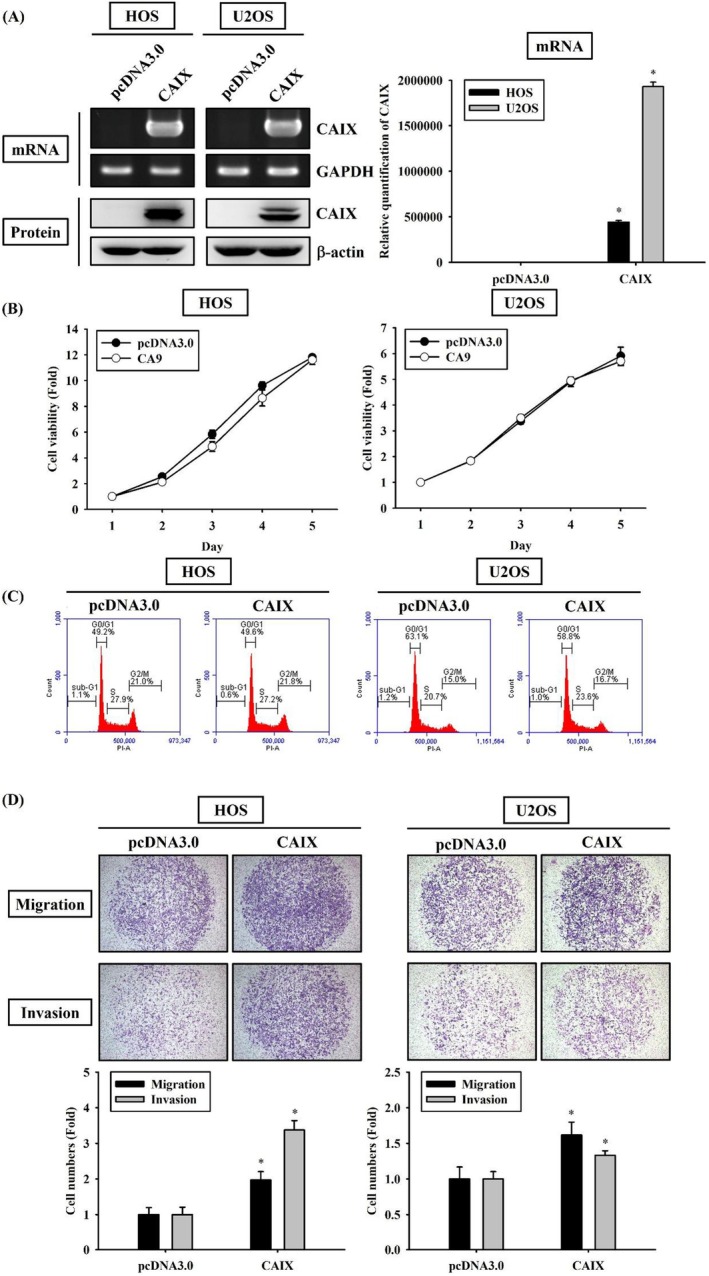
Overexpression of CAIX induces HOS and U2OS cell metastasis. The HOS and U2OS cells were transfected with empty vector control or CAIX expression plasmid. (A) The expression mRNA levels of CAIX in plasmid‐transfected HOS and U2OS cells were subjected to RT‐PCR. GAPDH was used as the loading control. Expression of CAIX was detected in cell mRNA by quantitative real‐time PCR. The value of CAIX was normalised to GAPDH levels. Cell lysates were analysed by western blot analysis to detect the expression levels of CAIX. β‐Actin was used as a loading control. (B) Growth curves of HOS and U2OS cells with or without CAIX overexpression were examined by MTT assay. (C) Cell cycle profiles of CAIX transfected HOS and U2OS cells were determined by flow cytometry. (D) The migratory and invasive activities of the HOS and U2OS cells were performed by Boyden chamber assay. Photos were taken using an inverted contrast light microscope under × 40 magnification. *p < 0.05 compared with control.

**FIGURE 3:**
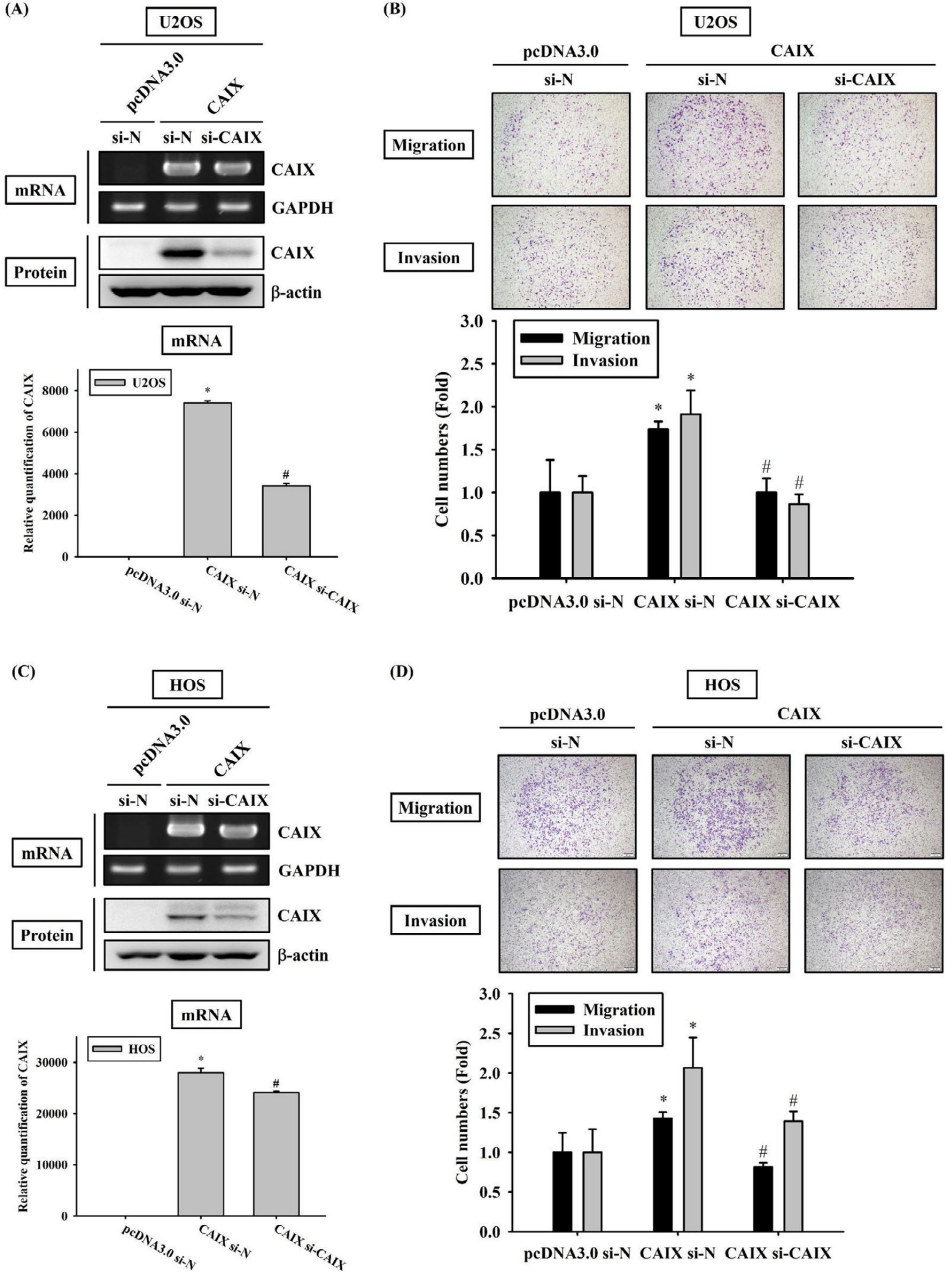
Knockdown CAIX represses of CAIX‐overexpressing U2OS cells metastasis. The CAIX‐overexpressing U2OS cells were transfected with negative siRNA or CAIX siRNA. (A) The expression mRNA levels of CAIX in siRNA‐transfected CAIX‐overexpressing U2OS cells were subjected to RT‐PCR. GAPDH was used as the loading control. Expression of CAIX was detected in cell mRNA by quantitative real‐time PCR. The value of CAIX was normalised to GAPDH levels. Cell lysates were analysed by western blot analysis to detect the expression levels of CAIX. β‐Actin was used as a loading control. (B) The migratory and invasive activities of the CAIX‐overexpressing U2OS cells transfected with siRNA were performed by Boyden chamber assay. Photos were taken using an inverted contrast light microscope under ×40 magnification. (C) The expression mRNA levels of CAIX in siRNA‐transfected CAIX‐overexpressing HOS cells were subjected to RT‐PCR. Expression of CAIX was detected in cell mRNA by quantitative real‐time PCR. Cell lysates were analysed by western blot analysis to detect the expression levels of CAIX. (D) The migratory and invasive activities of the CAIX‐overexpressing HOS cells transfected with siRNA were performed by Boyden chamber assay. Photos were taken using an inverted contrast light microscope under ×40 magnification. *p < 0.05 compared with control (pDNA3.0). # *p* < 0.05 when compared with osteosarcoma cells that were transfected with a CAIX overexpression vector.

To determine whether CAIX influences cell invasion and migration, HOS and U2OS cells were transfected with the pcDNA vector for mediating CAIX overexpression. We confirmed overexpression of CAIX mRNA and protein through RT‐PCR, real‐time PCR and western blotting in CAIX‐overexpressing U2OS cells, respectively (Figure 2A). To examine the cytotoxicity of CAIX, the MTT assay and flow cytometry were conducted. The results revealed that CAIX overexpression in HOS and U2OS cells for up to 5 days did not increase apoptosis, as evidenced by the nonsignificant changes in the cell cycle (Figure 2B,C). To verify the effects of CAIX on metastasis, Boyden chamber assays were performed using HOS and U2OS cells with or without CAIX overexpression to compare their migratory and invasive abilities. Intriguingly, CAIX overexpression significantly enhanced both migration and invasion in HOS and U2OS cells (Figure 2D). Moreover, the overexpression and knockdown of CAIX at both mRNA and protein levels were confirmed by RT‐PCR, real‐time PCR and western blotting in U2OS and HOS cells as well as in CAIX‐overexpressing U2OS and HOS cells (Figure 3A–D). As shown in Figure 3B,D, CAIX knockdown significantly impaired the migratory and invasive abilities of CAIX‐overexpressing U2OS and HOS cells. These results suggest that CAIX plays a critical role in promoting the migration and invasion of human OS cells.

### 3.3 CAIX Induces HSPA6 mRNA and Protein Expression in U2OS Cells

**FIGURE 4:**
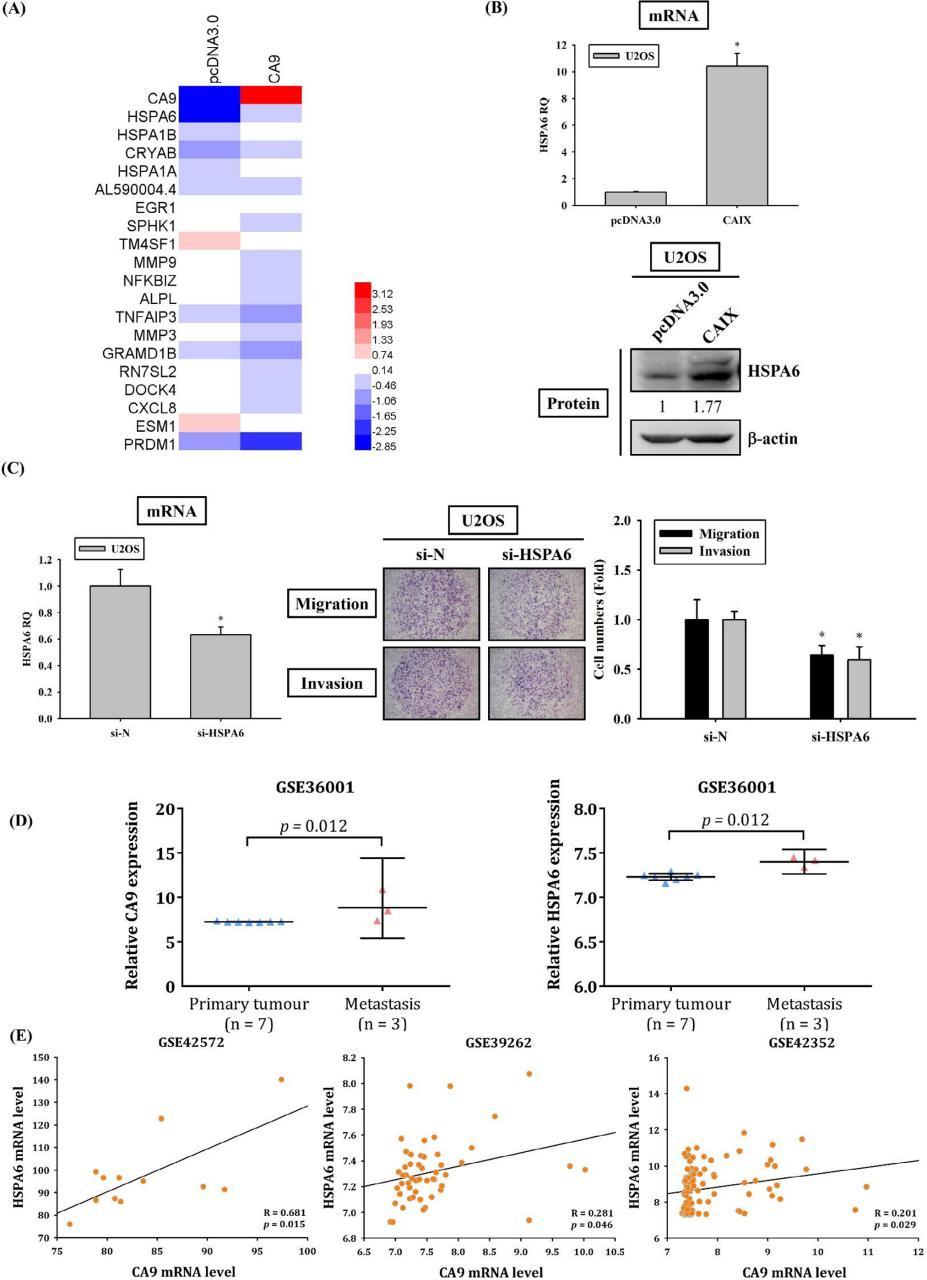
HSPA6 is a downstream target of CAIX and contributes to cell metastasis. (A) RNA sequencing for CAIX‐overexpressing U2OS cells. Heat map of the hierarchical clustering of 20 differentially expressed genes identified in U2OS cells after overexpressing the CAIX expression for 24 h. (B) Effects of CAIX on HSPA6 in U2OS cells. Real‐time PCR for HSPA6 mRNA expressions in U2OS cells after transfection with vectors containing a constitutively active CAIX cDNA were measured. Western blot analyses for the HSPA6 expression in U2OS cells after transfection with vectors containing a constitutively active CAIX cDNA were conducted. (C) Effects of HSPA6 on U2OS cells migration and invasion. Real‐time PCR for HSPA6 mRNA expressions in U2OS cells after siRNA directly against the HSPA6 expression were measured. Cell migration and invasion assays for U2OS cells after transfection with siRNA directly against the HSPA6 expression were measured. (D, E) To analyse the expression and correlation of CAIX and HSPA6 using the GEO database. *p < 0.05 compared with control.

To explore the mechanisms underlying the mediating effects of CAIX on the metastasis of OS cells through RNA sequencing technology, we conducted a transcriptomic analysis of U2OS cells transfected with a plasmid for 24 h to induce CAIX overexpression. In the RNA sequencing analysis of CAIX‐overexpressing U2OS cells, *HSPA6* was the most significantly upregulated gene in the CAIX‐overexpressing cells (Figure 4A). To validate the RNA sequencing findings for HSPA6, we conducted real‐time PCR and western blot analysis. The results revealed that CAIX‐overexpressing U2OS cells exhibited increased HSPA6 expression (Figure 4B).

### 3.4 HSPA6 Promotes U2OS Cell Migration and Invasion

To validate whether HSPA6 protein is the downstream molecule of CAIX and whether it influences cell migration and invasion, U2OS cells were directly transfected with siRNA for silencing HSPA6 expression. In real‐time PCR, HSPA6 mRNA expression was significantly decreased in HSPA6‐knocked‐down U2OS cells. Moreover, in the modified Boyden chamber assay, HSPA6 knockdown in U2OS cells significantly decreased cell migration and invasion (Figure 4C). We analysed CAIX and HSPA6 expression and their correlation in clinical specimens from the Gene Expression Omnibus (GEO) database. From the GEO database GSE36001, we found that CAIX expression and HSPA6 expression were higher in OS specimens with metastasis (Figure 4D). Additionally, from the GSE42572, GSE39262 and GSE42352 databases, a positive correlation was found between CAIX and HSPA6 expression (Figure 4E).

### 3.5 CAIX Induces HSPA6 Upregulation and Cell Migration and Invasion Through AMPK Signalling Pathways

**FIGURE 5:**
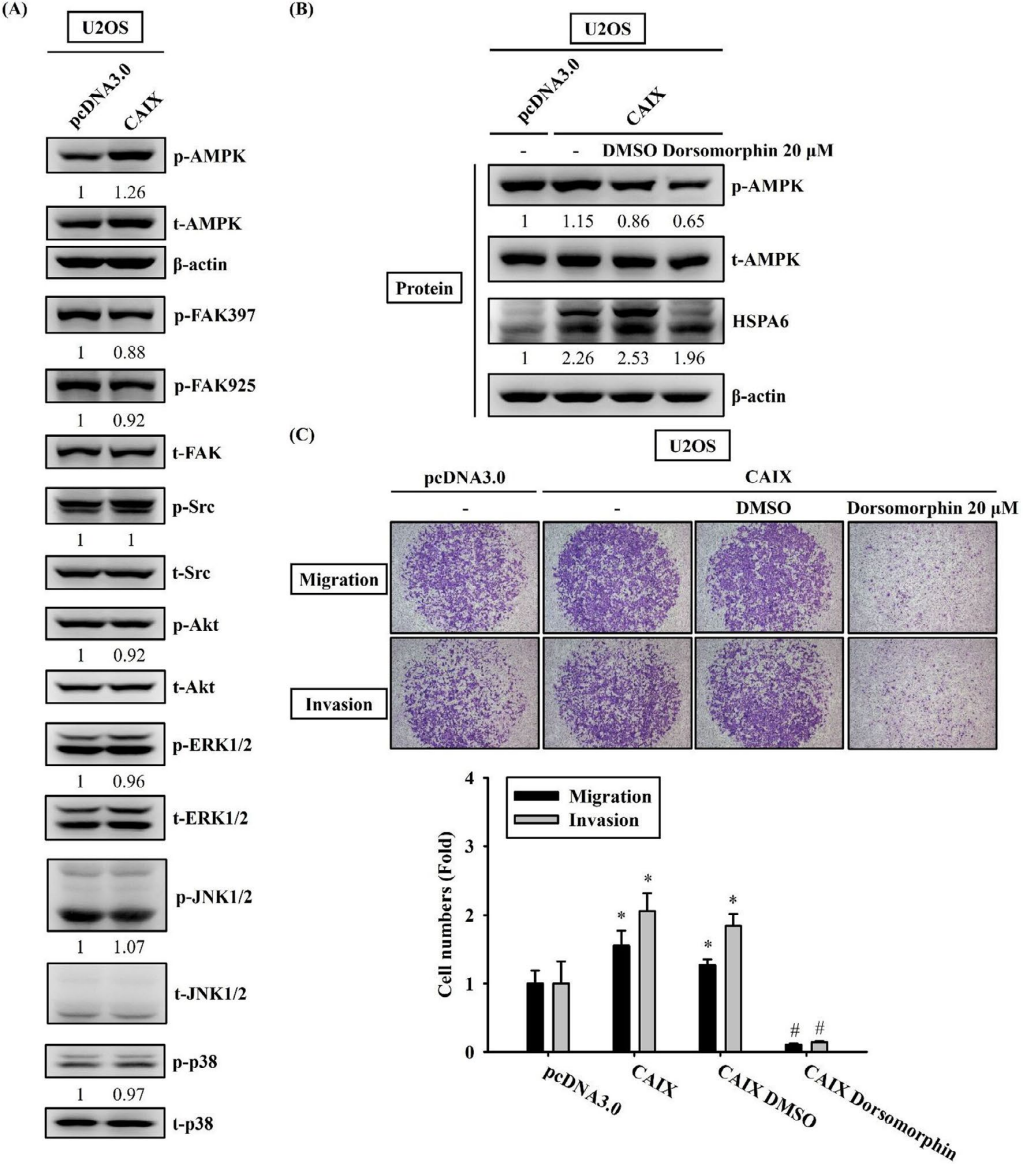
AMPK signalling pathway mediates CAIX‐induced HSPA6 expression and cell metastasis. (A) The levels of phosphorylated AMPK, FAK, Src, ERK, JNK, p38 and Akt in U2OS cells with or without CAIX expression were determined by western blot analysis of whole cell lysates. β‐actin was used as the loading control. (B) AMPK signalling pathway is crucial for CAIX‐induced HSPA6 up‐regulation, migration and invasion. U2OS cells with CAIX expression were treated with 20 μM of dorsomorphin for 18 h. Effects of dorsomorphin on AMPK and HSPA6 expression were examined by western blot analyses. β‐Actin served as the loading control. (C) Cells treated with 20 μM of dorsomorphin for 18 h were seeded into the Boyden chamber and incubated at 37°C for 24 h. Photos were taken using an inverted contrast light microscope under × 40 magnification. *p < 0.05 compared with control (pDNA3.0). # *p* < 0.05 when compared with osteosarcoma cells that were transfected with a CAIX overexpression vector.

To elucidate the signalling pathways involved in HSPA6 upregulation and migration and invasion induced by CAIX, we examined the activation status of signalling molecules in CAIX‐overexpressing U2OS cells. The results revealed the increased phosphorylation of AMPK in CAIX‐overexpressing U2OS cells (Figure 5A). To determine the functional roles of AMPK in HSPA6 expression and the invasive or migratory phenotypes of CAIX‐overexpressing U2OS cells, we used a specific inhibitor of AMPK, namely dorsomorphin. Dorsomorphin significantly suppressed AMPK phosphorylation and HSPA6 expression in CAIX‐overexpressing U2OS cells (Figure 5B). Additionally, dorsomorphin markedly attenuated CAIX‐induced migration and invasion of U2OS cells (Figure 5C). These results indicate that CAIX induces HSPA6 upregulation and migratory and invasive abilities in U2OS cells through activation of AMPK signalling pathways.

## 4 Discussion

In this study, we explored the overexpression of CAIX without cytotoxicity inducing cell migration and potential invasiveness in low CAIX expressions of HOS and U2OS cells. Using RNA sequencing technology, the upregulated protein of HSPA6 was intriguingly observed after overexpression of CAIX in U2OS cells. Researchers have examined CAIX and OS. Okuno and colleagues reported that patients with OS and high CAIX expression had a poor prognosis and that treatment with CAIX inhibitors under hypoxic conditions significantly inhibited cell proliferation and metastasis [33]. Perut and colleagues found that CAIX expression was significantly higher in OS cell lines than in bone marrow stromal cells. Moreover, CAIX inhibitors induced significant cytotoxicity in OS cells without affecting the cells' proliferation. CAIX activity is enhanced under hypoxic conditions and is regulated through cell cycle arrest and pH modulation in the cytoplasm and extracellular microenvironment. In mice, a CAIX inhibitor reduced tumour growth by inducing significant necrosis [34]. In this study, we found that CAIX promoted the metastasis of OS cells and increased the expression of HSPA6. We also discovered that inhibiting HSPA6 expression led to reduced metastasis of OS cells.

Heat shock proteins (HSPs) are induced under various stress conditions, such as heat, inflammation, UVB radiation and exposure to heavy metals, organic compounds, oxidative radicals and chemopreventive agents. HSPs play crucial functions in guiding proper folding, degradation and assembly of other proteins [35, 36, 37, 38, 39]. HSPs are classified into six families on the basis of their molecular size: the HSP100, HSP90, HSP70, HSP60, HSP40 and small HSP (15–30 kDa) families [40]. HSPs are involved in biological processes such as cell proliferation, cell death, apoptosis, immune response and oncogenesis [35, 36, 40]. HSPA6, also known as HSP70B, belongs to the HSP70 family and is responsible for the proper folding and activation of many proteins [41]. HSPA6 has been studied in human neuronal cells [42, 43, 44, 45, 46, 47, 48, 49] and cancer cell lines [36, 50]. Under normal conditions, HSPA6 expression is undetectable in most cells but is induced under severe stress conditions; thus, it may regulate the protective functions of cells [50, 51, 52]. Moreover, Hoter et al. reported that the expression of HSPA6 was upregulated under hypoxic conditions [53]. These findings are consistent with our results showing that the expression of CAIX and HSPA6 was upregulated in U2OS and HOS cells.

Studies have demonstrated that HSPA6 is involved in apoptosis and cell differentiation and plays a role in protecting differentiated human neuronal cells from cellular stress [54]. Moreover, HSPA6 expression is induced only after severe stress [55]. HSPA6 serves as a secondary responder to protein toxicity stress by directly binding to Apaf‐1 and inhibiting the assembly of functional apoptosomes, thus ensuring cell survival [56, 57]. Additionally, the reduced expression of HSPA6 in human colon cancer cell lines was found to lead to cell death through proteasome inhibition [58]. A study confirmed the association between high HSPA6 expression and the progression of atherosclerosis [59]. Studies have also indicated that HSPA6 expression is increased in hepatocellular carcinoma and breast cancer and that HSPA6 expression is associated with the early recurrence of hepatocellular carcinoma [60, 61]. Furthermore, one study found that HSPA6 enhances the inhibitory effects of garlic extract on the proliferation, migration and invasion of bladder cancer cells [62]. Thus, according to the literature, CAIX and HSPA6 are closely associated with the metastasis of certain cancers. Although some studies have been conducted on the role of CAIX in OS, no study has examined the role of HSPA6 in the metastasis of OS.

The AMPK signalling pathway has a versatile role in many cancers, including OS [63, 64]. For example, the activation of the AMPK signalling pathway can enhance the migratory and invasive properties of osteosarcoma cells by regulating the expression of matrix metalloproteinases [64]. Our study confirms that inhibiting AMPK phosphorylation significantly suppresses the metastasis of OS cell lines. However, the limitations of this study include the lack of an in vivo study; therefore, further studies are required to confirm whether the anti‐metastatic actions in vivo occur as those in vitro.

**FIGURE 6:**
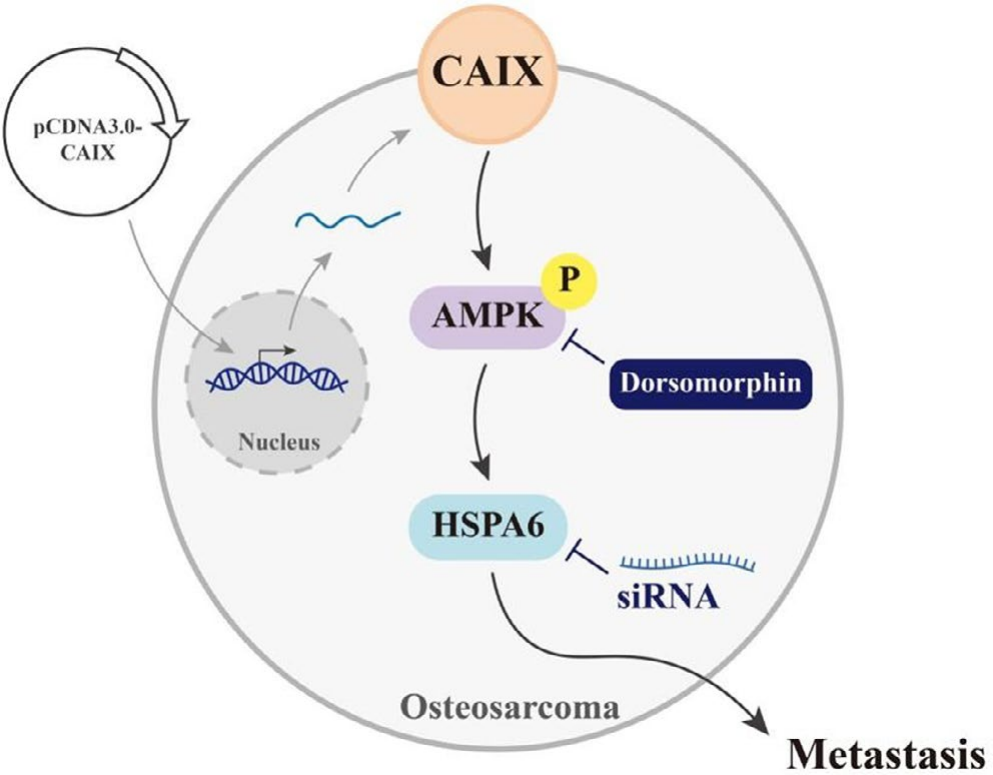
A schematic diagram illustrates that CA IX regulates HSPA6 expression through AMPK pathway activation. Overexpression of CAIX induces HSPA6 expression through phosphorylation of the AMPK pathway and induces the potential for metastasis in osteosarcoma cells.

In conclusion, CAIX promotes the metastasis of OS cell lines by increasing HSPA6 expression through the AMPK signalling pathway (Figure 6). The observations made in the present study suggest that CAIX has a novel function in promoting the migration and invasion of cancer cells. Thus, it is a potential therapeutic target for OS.

## Author Contributions

**Jia‐Sin Yang:** conceptualization (equal), data curation (equal), writing – original draft (equal), writing – review and editing (equal). **Chia‐Hsuan Chou:** conceptualization (equal), data curation (equal), writing – original draft (equal). **Yi‐Hsien Hsieh:** data curation (equal), software (equal), software (equal). **Chih‐Hsin Tang:** data curation (equal), software (equal). **Ko‐Hsiu Lu:** conceptualization (equal), data curation (equal), methodology (equal), writing – original draft (equal), writing – review and editing (equal). **Shun‐Fa Yang:** conceptualization (equal), methodology (equal), writing – original draft (equal), writing – review and editing (equal).

## Conflicts of Interest

The authors declare no conflicts of interest.

## Data Availability

The data that support the findings of this study are available from the corresponding author upon reasonable request.
